# Oxygen Vacancy Modification MIL-125(Ti) Promotes CO_2_ Photoreduction to CO with Near 100% Selectivity

**DOI:** 10.3390/ma18061343

**Published:** 2025-03-18

**Authors:** Hangmin Xu, Hao Song, Xiaozhi Wang, Xingwang Zhu

**Affiliations:** 1 College of Environmental Science and Engineering, Institute of Technology for Carbon Neutralization, Yangzhou University, Yangzhou 225009, China; xhm@zxw.ac.cn (H.X.); sh@zxw.ac.cn (H.S.); 2 Jiangsu Collaborative Innovation Center for Solid Organic Waste Resource Utilization, Nanjing 210095, China

**Keywords:** MIL-125(Ti), oxygen vacancy, Ti^3+^ centers, CO_2_ photoreduction

## Abstract

The substantial release of industrial carbon dioxide has been identified as a key factor in the development of various environmental issues. In addressing these concerns, the utilization of photocatalytic technology for carbon reduction has garnered significant attention. The disadvantage of CO_2_ photoreduction is the problem of product yield and selectivity. It is known that MIL-125(Ti) with a high specific surface area (S_BET_) possesses more active sites using Ti as a node. The calcination of MIL-125(Ti) in a reducing atmosphere has been shown to introduce oxygen vacancies (O_V_), thereby enhancing the material’s surface and internal pores. This process has been demonstrated to result in a significant increase in the S_BET_ and an enhancement of the Ti^3+^/Ti^4+^ ratio. The increased Ti^3+^ centers have been found to improve the material’s reducing properties. The results demonstrate that the O_V_-rich MIL-125-2H material exhibits the high-performance and highly selective photoreduction in CO_2_.

## 1. Introduction

The issue of industrial carbon dioxide (CO_2_) emissions represents a significant challenge to global environmental improvement [1,2,3]. The utilization of advanced technology for the mitigation of carbon emissions has emerged as a primary focus for scholars in the field. The development of artificial photocatalytic technology in this field has been a long-standing endeavor. Nevertheless, the implementation of photocatalytic technology remains encumbered by several challenges, including light absorption, CO_2_ adsorption and desorption, and catalyst stability [4,5].

Metal–organic frameworks (MOFs) are structures that possess internal porosity supported by a metallic backbone, formed by the coordination of metal ions/clusters and organic ligands [6]. The attention that is now being paid to MOFs in a variety of fields, such as gas adsorption, is due to their multifunctional structure and high specific surface area [7]. In particular, nano-sized MOFs have been shown to expose a greater number of active sites, thereby enhancing their performance [8,9]. It has been established that the size of MIL-125(Ti) is in the nanoscale, which has been demonstrated to promote an increase in the specific surface area and active sites of the MOFs, thereby facilitating gas photocatalytic activation. Concurrently, the elevated specific surface area of MIL-125(Ti) facilitates the promotion of Ti-O-C connections, thereby enhancing the capacity for CO_2_ adsorption on the MIL-125(Ti) surface and facilitating CO_2_ activation [10]. The adsorption fixation of CO_2_ by nanoscale titanium-based MOF has been reported in the literature, suggesting that MIL-125(Ti) has the potential to act as a photocatalytic activator of CO_2_ [11,12]. However, the further modification of MIL-125(Ti) to enhance its intrinsic properties and effectively improve its gas adsorption capacity at the interface, increase the active sites, and shorten the diffusion path of the guest molecules is challenging.

In recent years, there has been a growing body of research focusing on the impact of oxygen vacancies in MOFs on electron transfer in semiconductors [13,14]. These O_V_ are known to act as sites for immobilizing metal atoms, facilitating acid–base interactions, and enabling uniform loading of donors/acceptors [15]. In light of these observations, we propose a hypothesis that the introduction of O_V_ around the Ti metal of MIL-125(Ti) allows the excited state electrons to first accumulate within O_V_ and, later, further transfer to the Ti sites to enhance charge separation and transfer [16]. Sun et al. demonstrated that, by introducing oxygen vacancies, the concentration of Ti^3+^ in the active site could be regulated, thereby affecting the electron transfer pathway during its photocatalytic nitrogen fixation and ammonia production [17]. However, the presence of excess O_V_ has been shown to inhibit the separation of photogenerated carriers, which has a detrimental effect on the catalytic performance of the catalysts [18]. Therefore, the introduction of an appropriate amount of O_V_ in MIL-125(Ti) for the application of photocatalytic reduction in CO_2_ is particularly important.

In this study, MOFs comprising Ti as the metal node were synthesized and calcined under a reducing gas atmosphere. This process resulted in an augmentation of the specific surface area (S_BET_) of MIL-125(Ti) without compromising the structural integrity. Concurrently, it facilitated a reduction in the proportion of Ti^4+^ to Ti^3+^, thereby enhancing the reduction capacity of the Ti sites. Concurrently, the oxygen vacancies that were introduced promoted the aggregation of nearby electrons to the Ti sites and further transferred them to the adsorbed CO_2_. Incorporating theoretical calculations reveals that the O_V_ introduced is instrumental in reducing the energy barrier associated with the activated CO_2_ step of the MIL-125-2H, thereby enhancing its efficiency. In conclusion, the modified MIL-125-2H reduced CO_2_ to CO in yields of up to 771.22 μmol g^−1^ h^−1^, as well as the selectivity of up to near 100%. The investigation revealed that oxygen-rich vacancies (O_V_-rich) and a high specific surface area may play a significant role in the photocatalytic CO_2_ reduction process.

## 2. Experimental Section

### 2.1. Chemical Reagent

Terephthalic acid (C_8_H_6_O_4_, ≥99%) and tetra-butyl ortho-titanate (Ti(OC_4_H_9_)_4_, ≥99%) were purchased from Aladdin Biochemical Technology Co., Ltd. (Shanghai, China). N,N-dimethylformamide (DMF, ≥99.5%) and methanol (CH_3_OH, ≥99.5%) were purchased from Sinopharm Chemical Reagent Co., Ltd. (Shanghai, China) These reagents were purchased directly for use and have not been handled in any way. Ultrapure water (18.25 MΩ/cm) was prepared by a laboratory ultrapure water machine (YL-400BU, Shenzhen YL Electronics Co., Ltd., Shenzhen, China).

### 2.2. Preparation of MIL-125(Ti)

The preparation of MIL-125(Ti) was conducted in accordance with methods previously documented in the extant literature [17]. The C_8_H_6_O_4_ (1.5 g, 9 mmol) was dissolved in 27 mL of DMF and ultrasonicated for a period of 5 min until complete dissolution. Concurrently, Ti(OC_4_H_9_)_4_ (0.78 mL, 2.25 mmol) was gradually added to 3 mL of methanol under vigorous stirring to yield a clear solution. The methanol solution containing titanate was subsequently transferred to the organic linker that has been previously described. Following a 30 min stirring period, a gradual transition of the color of the mixture from yellow to milky white was observed. Subsequently, the milky mixture was transferred to a flask and heated in an oil bath at 130 °C for 24 h. After cooling to room temperature, the suspension was subjected to centrifugation at 6000 rpm for 3 min. The precipitate was collected and subsequently washed several times with DMF in order to remove unreacted organic ligands. Thereafter, the unreacted Ti ions were removed by washing with methanol, after which the precipitate was collected by centrifugation. The solid was then dried under vacuum at 80 °C for 5 h to yield a white solid with a yield of approximately 0.5 g.

### 2.3. Preparation of MIL-125-xH

The MIL-125-xH was prepared by subjecting MIL-125(Ti) to heat treatment at 250 °C for various durations under the protection of a mixture of Ar and H_2_ (Ar 95%, H_2_ 5%). The heating rate was set to 5 °C/min, the gas flow rate was set to 0.1 L/min, and the samples were allowed to cool naturally to room temperature before the aeration process was halted. The duration of the heat treatment was 0.5, 2, and 5 h and corresponded to MIL-125-0.5H, MIL-125-2H, and MIL-125-5H, respectively.

### 2.4. Photocatalytic Measurements

A quantity of 10 mg of photocatalyst was added to a photoreactor, together with 4 mL of ultrapure water, 2 mL of lactic acid, and 6 mL of acetonitrile. The mixture was then sonicated in order to ensure uniform dispersion. The temperature of the photoreactor was controlled at 10 °C by circulating cooling water, with the aim of preventing the decomposition of the solution. The reaction was continuously stirred with a magnetic stirrer at 500 rpm. The photoreactor was then connected to the photocatalytic carbon dioxide reduction system, after which the entire system was evacuated to vacuum using a vacuum pump. The system was then charged with 20 kPa of high-purity CO_2_, and the lines were rinsed and vacuumed to eliminate other residual gases. Finally, high-purity CO_2_ was reintroduced until a pressure of 80 kPa was reached. A 300 W xenon lamp (PLS-SEX300, Beijing Perfectlight, Beijing, China) was utilized as a light source during the reaction, and at the hourly interval, 1 mL of gas was withdrawn from the reaction system into a gas chromatograph (GC9790II) to detect the products (5 h as a reaction cycle).

## 3. Results and Discussion

**Figure 1:**
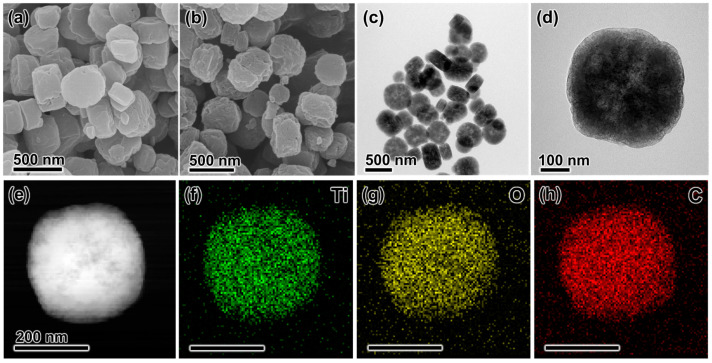
The SEM images of (**a**) MIL-125(Ti) and (**b**) MIL-125-2H. (**c**) TEM image of MIL-125-2H. (**d**) The HR-TEM image of MIL-125-2H. (**e**) STEM image and (**f**–**h**) elemental distribution map of MIL-125-2H.

The original MIL-125(Ti) was a strong skeleton synthesized using Ti as the metal junction and terephthalic acid as the organic ligand [19]. In this study, a low-temperature calcination approach was employed to introduce oxygen vacancies in the vicinity of the metal nodes without compromising the integrity of the MIL-125(Ti) backbone. Prior to calcination, the morphology of MIL-125(Ti) as observed under the scanning electron microscope (SEM) exhibited a round cake-like shape and a relatively smooth surface (Figure 1a). Following calcination at a low temperature of 250 °C for different durations, the skeletons of MIL-125-0.5H (Figure S1a), MIL-125-2H (Figure 1b), and MIL-125-5H (Figure S1b) remained unchanged, as evidenced by SEM analysis. However, an increase in the number of pores on the catalyst surface was observed, indicating that calcination led to an enhancement in the S_BET_ of MIL-125-xH. In the transmission electron microscope (TEM), the size of MIL-125-2H was found to be approximately 300–500 nm (Figure 1c). As demonstrated in Figure 1d (high resolution- TEM, HR-TEM), no additional impurities were identified on MIL-125-2H, and the interior was found to be sustained by a skeleton rather than a solid structure. In the elemental distribution map (Figure 1e–h), Ti, O, and C demonstrate a uniform distribution.

**Figure 2:**
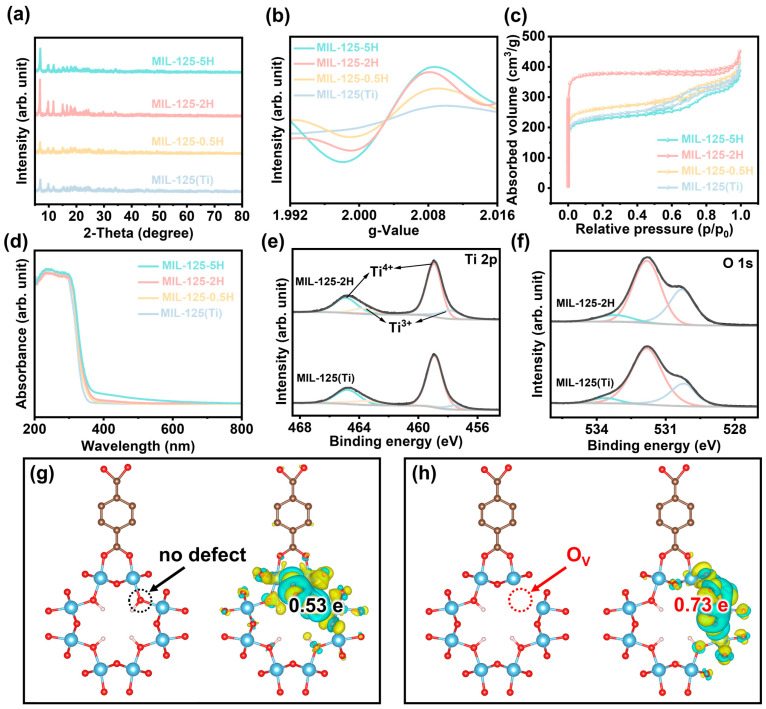
(**a**) XRD profiles, (**b**) EPR spectra, (**c**) N_2_ adsorption and desorption curves, and (**d**) UV-vis DRS spectra of samples. The high-resolution XPS spectrum of (**e**) Ti 2p and (**f**) O 1s. The Bader charge of (**g**) MIL-125(Ti) and (**h**) MIL-125-2H. The green, red, yellow, and blue curves in panels (**e**,**f**) are the XPS splitting curves in MIL-125(Ti) and MIL-125-2H. (**e**,**f**) show the upper part of the curves as MIL-125-2H and the lower part of the curves as MIL-125(Ti). Therefore, to avoid confusion, we have changed the nomenclature of MIL-125(Ti) and MIL-125-2H in panels (**e**,**f**) to black.

To explore the crystal structure of MIL-125-xH (x = 0.5, 2, 5), X-ray diffraction (XRD) was employed. As demonstrated in Figure 2a, the diffraction peaks of MIL-125-xH remain largely unchanged in comparison to those of MIL-125(Ti), suggesting that the phase structure of MIL-125-xH remains unchanged following calcination [20,21]. The crystalline size was calculated based on XRD, and as demonstrated in Table S1, the crystalline size increased in the calcined samples but decreased after an extended period of calcination. However, it is noteworthy that the crystalline size of all the samples was below 100 nm, which is in the nanoscale. This facilitates an increase in the specific surface area of the sample and improves the capture of CO_2_ [22]. In order to comprehend the concentration of O_V_ in MIL-125-xH, electron paramagnetic resonance (EPR) analysis (Figure 2b) was conducted, revealing that oxygen vacancies were not detected in MIL-125(Ti) prior to calcination. Subsequent to calcination, the concentration of oxygen vacancies exhibited a gradual enhancement with increasing calcination time [23]. In the N_2_ adsorption–desorption test, the S_BET_ of MIL-125(Ti) was 910.05 m^2^/g. In comparison, the S_BET_ of MIL-125-xH increased gradually with the increase in calcination time, which was mainly due to the increase in calcination time, which increased the pore space of the sample. However, when the calcination duration reached 5 h, the S_BET_ of MIL-125-5H was only 882.54 m^2^/g. This was due to the long calcination time, which led to the partial decomposition of organic ligands and blocked the pores in the sample. The MIL-125-2H prepared after 2 h of calcination had the largest specific surface area (1481.29 m^2^/g), providing more active sites for CO_2_ reduction. Therefore, the functional groups in the samples were analyzed using Fourier transform infrared (FTIR) spectroscopy. As demonstrated in Figure S2, MIL-125(Ti) and MIL-125-xH exhibit analogous characteristic peaks. The range of 400–800 cm^−1^ is attributed to the stretching vibration of (O-Ti-O). The 1380–1654 cm^−1^ range is attributed to the stretching vibration of the backbone O-C-O and C=O, which is characteristic of the carboxylate band, thus confirming the presence of carboxylate linkers in the material [17,21]. Meanwhile, these characteristic peaks did not change with the increase in calcination duration. However, in the context of Raman spectroscopy (Figure S3), the characteristic peaks of MIL-125-xH appeared to diminish with the prolongation of the calcination duration, and in particular, almost no signal was detected in MIL-125-5H. Combined FTIR and Raman spectroscopy has been demonstrated to show that a small amount of ligand decomposition occurs in MIL-125-5H.

In the UV-visible diffuse reflectance spectra (UV-vis DRS), the MIL-125-xH light absorption edge demonstrates a slight redshift with increasing calcination time, which is primarily attributable to the deepening of the color of MIL-125-xH as a consequence of calcination, thereby augmenting its light absorption range [17]. Subsequently, the light absorption capacity of the samples was calculated by constructing models for MIL-125(Ti) and MIL-125-2H. As demonstrated in Figure S4a,b, within the range of ~370–400 nm, MIL-125-2H exhibits a more pronounced light absorption capacity in comparison to MIL-125(Ti), thereby substantiating the assertion that the incorporation of O_V_ extends the light absorption and establishes the basis for efficient photoexcitation of electrons [24]. Consequently, the density of states for the samples was subsequently calculated and compared to that of MIL-125(Ti). MIL-125-2H was found to exhibit a higher density of states at the Fermi level (Figure S5a,b). The introduction of O_V_ was found to increase the semiconductor intrinsic semi-conductivity and facilitate electron transfer [25]. X-ray photoelectron spectroscopy (XPS) was applied to reveal the local electronic structure and valence states of the prepared samples. Three major peaks belonging to Ti, O, and C were detected in the full XPS spectrum (Figure S6). In the XPS spectrum of Ti 2p (Figure 2e), the peaks with binding energies at positions 464.90 eV and 458.95 eV are attributed to Ti^4+^, while the peaks with binding energies at positions 463.65 eV and 457.50 eV are ascribed to Ti^3+^ [26]. However, the peak area of the Ti^3+^ in MIL-125-2H increased significantly after 2 h of calcination. This is due to the increase in oxygen vacancies, which partially convert Ti^4+^ to Ti^3+^. As shown in Table S2, the ratio of Ti^3+^/Ti^4+^ increased to 0.30 for MIL-125-2H compared to 0.23 for MIL-125(Ti). Subsequently, the change in O_V_ content was further determined from the XPS spectrum of O 1s. As demonstrated in Figure 2f, the binding energies situated at 530.20, 531.85, and 533.65 eV are attributed to adsorbed oxygen, oxygen vacancies, and the lattice oxygen of MIL-125(Ti), respectively [27]. The peak intensity of the oxygen vacancies belonging to MIL-125-2H was slightly enhanced in comparison with MIL-125(Ti), indicating that calcination did enhance the concentration of O_V_, which is consistent with the Ti^4+^/Ti^3+^ conversion in MIL-125-2H and the results of the EPR tests.

Based on the structural analysis, theoretical computational models of MIL-125(Ti) without defects and MIL-125-2H containing O_V_ near the Ti nodes were constructed, as shown in Figure 2g,h. The results of the calculation of the Bader charge of CO_2_ adsorbed at the sample interface (Figure 2g,h) demonstrate that, in the absence of O_V_, electrons are more likely to accumulate at the Ti node. Concurrently, a greater number of electrons are transferred to CO_2_ by MIL-125-2H (0.73 e) in comparison to MIL-125(Ti) (0.53 e), thereby promoting CO_2_ photoreduction [25,28].

**Figure 3:**
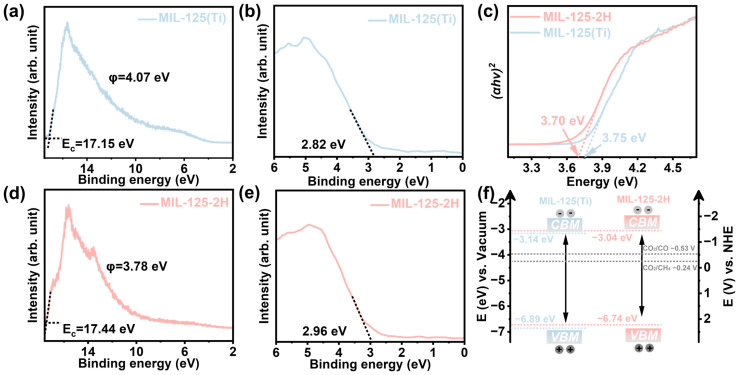
The UPS spectrum and VB-XPS of (**a**,**b**) MIL-125(Ti) and (**d**,**e**) MIL-125-2H. (**c**) The band gap diagram and (**f**) the positions of band structure of samples.

In order to analyze whether the band structure of the samples meets the thermodynamic requirements for photocatalytic reduction in CO_2_, ultraviolet photoelectron spectroscopy (UPS) and valence band-XPS (VB-XPS) were employed. As demonstrated in Figure 3a,d, the UPS cut-off edges (E_C_) of MIL-125(Ti) and MIL-125-2H are 17.15 eV and 17.44 eV, respectively. The work functions of these two materials are 4.07 eV and 3.78 eV, as calculated based on *φ* = *hv* − E_C_ (*hv* = 21.22 eV) [29]. The results of the VB-XPS tests (Figure 3b,e) demonstrate that the VB of MIL-125(Ti) and MIL-125-2H are 2.82 eV and 2.96 eV, respectively. Consequently, the valence band maximum (VBM, E_VB, Vac_) of the two relative to the vacuum level is calculated to be −6.89 eV and −6.74 eV, respectively. The potentials of the samples were converted, according to the standard hydrogen electrode (E_VB,NHE_ = −E_VB,Vac_ − 4.44) [30]. The band gaps of MIL-125(Ti) and MIL-125-2H are 3.75 eV and 3.70 eV, respectively, as determined by the Tauc plots of the UV-vis DRS transformations. The conduction band minimum (CBM) was determined through calculation of the band gap, and the band structure of the sample was found to satisfy the thermodynamic requirements for CO_2_ photoreduction (Figure 3f). Concurrently, the CBM of MIL-125-2H is higher than that of MIL-125(Ti), which renders MIL-125-2H more capable of reduction than MIL-125(Ti).

**Figure 4:**
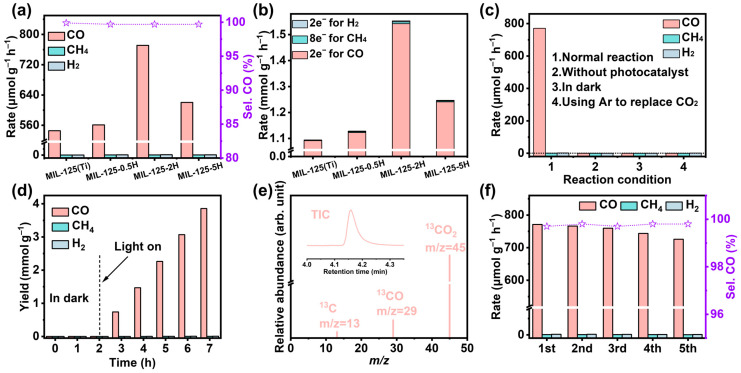
(**a**) Comparison of the rate of photocatalytic reduction in CO_2_ products from samples and the selectivity of CO. (**b**) Number of molar electrons consumed in reaction. (**c**) Comparison of CO_2_ photoreduction performance under different reaction conditions. (**d**) Gas products of CO_2_ photoreduction as a function of time. (**e**) GC-MS spectra of ^13^CO_2_ photoreduction products by MIL-125-2H. (**f**) Stability testing of catalyst.

The CO_2_ photoreduction properties of the samples under investigation were evaluated by performing the reaction in a system of 6 mL acetonitrile, 4 mL H_2_O to provide the proton source, and 2 mL triethanolamine as a sacrificial agent. The reaction was completed in the μGAS1000 system (Figure S7). The CO_2_ reduction rate test results (Figure 4a) demonstrated that the calcined MIL-125-xH facilitated the reduction in CO_2_ to CO at a higher rate than MIL-125(Ti) (545.54 μmol g^−1^·h^−1^). Among the samples, MIL-125-2H (771.22 μmol g^−1^·h^−1^) exhibited the highest rate of reduction to CO, which is hypothesized to be attributable to its augmented S_BET_, which provides an increased number of active sites and facilitates greater electron transfer from Ti^3+^ active sites to CO_2_. It is also noteworthy that, despite the presence of CH_4_ and H_2_ in the products, all samples exhibit the selective conversion of up to near 100% for CO. The calculation of the number of moles of consumed effective photogenerated electrons throughout the reaction indicates that almost all of the consumed photogenerated electrons are involved in the photoreduction in CO_2_ to CO, which is consistent with the gas product selectivity results (Figure 4b). A series of comparative experiments were conducted in order to explore the factors that influence the reaction process. As demonstrated in Figure 4c, in the absence of a catalyst addition to the reaction system, no gas was produced. Similarly, in the absence of light, no gas was produced. No production of CO and CH_4_ was observed under dark conditions for the initial 2 h; however, a linear increase in CO production was immediately evident upon the activation of the lights (Figure 2d). These findings indicate that the complete reaction must occur in the presence of a catalyst and must be catalyzed by light, both of which are essential components. Following the substitution of Ar for CO_2_, it was determined that a negligible amount of H_2_ (1.75 μmol g^−1^ h^−1^) was produced. This finding indicates that the C source of CO and CH_4_ is derived from the reduction in CO_2_, rather than from the decomposition of C in the catalyst or from the decomposition of organic reagents in solution. This outcome serves to reinforce the conclusion that only a minimal number of molar photogenerated electrons are implicated in the hydrogen evolution reaction (HER). In order to establish that CO and CH_4_ are derived from CO_2_, ^12^CO_2_ gas was replaced by ^13^CO_2_ gas (Figure 4e). A high-intensity signal with a m/z = 29 was detected in the GC-MS. This signal is believed to be ^13^CO. However, no signal was detected for ^13^CH_4_, which was attributable to the low yield of ^13^CH_4_ [31,32]. Consequently, it can be concluded that CO is indeed produced by CO_2_ activation. Finally, an investigation was conducted into the stability of MIL-125-2H. As demonstrated in Figure 4f, it was determined that there was no substantial decline in its activity after five cycles. Post-reaction SEM (Figure S8) revealed that the backbone of MIL-125-2H remained unaltered and did not undergo collapse. Subsequently, an investigation was conducted into the ratio of Ti^3+^/Ti^4+^ in MIL-125-2H following the stability test. The results, as illustrated in Figure S9 and Table S3, demonstrated that there was no alteration in the ratio of Ti^3+^/Ti^4+^ peak areas. This finding suggests that MIL-125-2H exhibits excellent stability. The performance of CO_2_ photoreduction in the present study was subsequently compared with a similar study. It emerged that the introduction of O_V_ in MIL-125(Ti) resulted in superior performance (Table S4).

**Figure 5:**
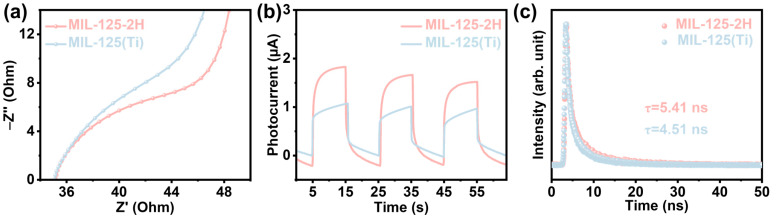
(**a**) The EIS spectra, (**b**) the transient photocurrent response, and (**c**) the TR-PL spectra of MIL-125(Ti) and MIL-125-2H.

Photogenerated electron transport properties were investigated by means of a series of sample photoelectric property tests. As shown in Figure 5a, MIL-125-2H exhibits electrochemical impedance spectroscopy (EIS) radius in comparison to MIL-125(Ti), indicating its enhanced efficacy in facilitating photogenerated electron transfer [33]. Subsequently, the photocurrent test revealed that MIL-125-2H exhibited a photocurrent of up to ~1.80 μA under light excitation, while MIL-125(Ti) demonstrated a maximum of ~1.0 μA. This finding suggests that MIL-125-2H is able to promote more photogenerated electron transfer than MIL-125(Ti) [34]. The steady-state fluorescence lifetime (PL) measurements demonstrate that the PL peak intensity of MIL-125-2H is considerably lower than that of MIL-125(Ti) at an excitation wavelength of 350 nm (Figure S10). This finding indicates that the introduction of oxygen vacancies has resulted in a substantial suppression of carrier complexation in the sample [35]. Next, the transient photoluminescence (TR-PL) lifetime of the photogenerated electrons transferred was examined. As demonstrated in Figure 5c, the TR-PL lifetime of MIL-125-2H (5.41 ns) is greater than that of MIL-125(Ti) (4.51 ns), which suggests a higher utilization of photogenerated electrons in MIL-125-2H [36,37]. Finally, the surface photovoltage of the samples was tested, and as demonstrated in Figure S11, compared to MIL-125(Ti), MIL-125-2H has a significant potential difference at 310–370 nm because of the presence of O_V_, which promotes the enrichment of electrons on O_V_ and their migration towards the Ti sites. The combined photoelectronic characterization demonstrates that the incorporation of O_V_ substantially hinders the photogenerated carriers from undergoing bulk and surface phase complexation on MIL-125-2H. Furthermore, the introduction of O_V_ enhances the lifetime of photogenerated carriers and facilitates the attainment of a higher photogenerated carrier separation efficiency in MIL-125-2H.

**Figure 6:**
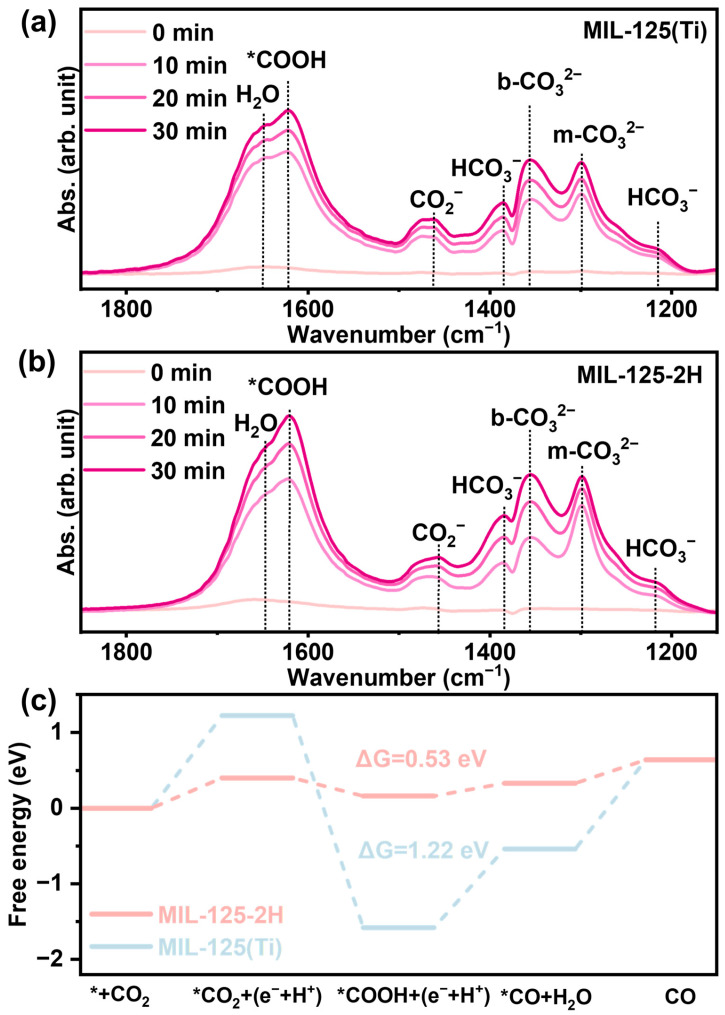
The in situ FTIR spectra under CO_2_ reaction conditions with increasing light time of (**a**) MIL-125(Ti) and (**b**) MIL-125-2H. (**c**) Calculation of the Gibbs free energy for the activation of CO_2_ to CO by MIL-125(Ti) and MIL-125-2H.

The present study employed in situ characterization techniques in conjunction with DFT calculations to explore the mechanism of the dynamic photoreduction process of CO_2_. The present study investigates the simulation of MIL-125(Ti) and MIL-125-2H photocatalytic activation of CO_2_ for in situ Fourier transform infrared spectroscopy (FT-IR) testing. As shown in Figure 6a,b, the wavenumbers at ~1298, ~1355, ~1462, ~1621, and ~1648 cm^−1^ correspond to m-CO_3_^2−^, b-CO_3_^2−^, CO_2_^−^, *COOH, and H_2_O, respectively. The wavenumbers at ~1215 cm^−1^ and ~1385 cm^−1^ correspond to HCO_3_^−^ [38,39]. These belong to the intermediates of CO_2_ reduction to CO, with no intermediates belonging to the CH_4_ intermediates. Meanwhile, the peaks of these intermediates were significantly enhanced with increasing light duration. The peaks belonging to H_2_O are also significantly enhanced, which is caused by the continuous decomposition of H_2_O to provide more proton sources. Compared with MIL-125(Ti), the intensity of the in situ FT-IR peaks of MIL-125-2H was stronger, indicating that MIL-125-2H has a greater ability to reduce CO_2_ to CO [40].

In consideration of the results obtained from in situ infrared spectroscopy, the following pathways for the photoreduction in CO_2_ can be identified:

(1) $${CO}_{2}+*\to *\;{CO}_{2}$$

(2) $${*\;CO}_{2}+H^{+}+e^{-}\to *\;COOH$$

(3) $$*\;COOH+H^{+}+e^{-}\to *\;CO+H_{2}O$$

(4) ∗ CO→CO↑+ ∗

As depicted in Figure 6c (* represents photocatalyst), the key rate steps on MIL-125(Ti) and MIL-125-2H are identified as CO_2_ adsorption and CO desorption at the interface with ∆G values of 1.22 eV and 0.53 eV, respectively. The presence of O_V_ in MIL-125-2H leads to a substantial reduction in the overall activation energy barriers and the formation energies of key intermediates [24]. Consequently, the strategic addition of O_V_ can greatly increase activity by lowering the energy barrier required for the overall reaction.

## 4. Conclusions

In the present study, an O_V_-rich strategy was developed, designed, and introduced on MIL-125(Ti). The introduction of O_V_ resulted in an increase in the specific gravity of Ti^3+^/Ti^4+^ on MIL-125-2H and the reduction capacity of Ti^3+^, thereby providing a greater number of active sites for CO_2_ photoreduction. The presence of O_V_ has been shown to facilitate the transfer of electrons from the Ti site on MIL-125-2H to CO_2_, in addition to lowering the activation energy barrier of CO_2_. In consideration of the aforementioned advantages, MIL-125-2H demonstrated a reduction in CO_2_ to CO with a high yield and high selectivity of 771.22 μmol g^−1^ h^−1^ and near 100%, respectively. The findings of the study culminated in the synthesis of an efficient and stable catalyst, thus providing a novel strategy for the application of vacancy engineering to artificial carbon photoreduction.

## Data Availability

The original contributions presented in this study are included in the article/Supplementary Materials. Further inquiries can be directed to the corresponding authors.

## Supplementary Materials

The following supporting information can be downloaded at: https://www.mdpi.com/article/10.3390/ma18061343/s1, Figure S1: The SEM images of (a) MIL-125-0.5H and (b) MIL-125-5H. Figure S2: FTIR spectroscopy of the sample. Figure S3: Raman spectroscopy of the sample. Figure S4: Theoretical calculation of light absorption spectrum of (a) MIL-125(Ti) and (b) MIL-125-2H. Figure S5: The density of state of (a) MIL-125(Ti) and (b) MIL-125-2H. Figure S6: The full XPS spectrum of MIL-125-0.5H and MIL-125-5H. Figure S7: CO_2_ photocatalytic reaction system. Figure S8: SEM image of MIL-125-2H after stability test. Figure S9: The XPS spectrum of MIL-125-2H after stability test. Figure S10: The PL spectrum of MIL-125(Ti) and MIL-125-2H. Figure S11: The SPV image of MIL-125(Ti) and MIL-125-2H. Table S1: The crystallite size of the sample. Table S2: Ratio of Ti^3+^ and T^4+^ content. Table S3: The ratio of Ti^3+^/Ti^4+^ before and after the MIL-125-2H reaction Table S4: Comparison of the performance of this work with similar literature on photocatalytic reduction of CO_2_ to CO. References [41,42,43,44,45,46,47] have been cited in Supplementary Materials file.
