# Supplementary material for: Oxygen Vacancy Modification MIL-125(Ti) Promotes CO2 Photoreduction to CO with Near 100% Selectivity
Source: Materials (Basel). 2025 Mar 18;18(6):1343. doi: 10.3390/ma18061343 (PMC11943590; doi:10.3390/ma18061343)
Supplement: Supplementary file 1 [file materials-18-01343-s001.zip › materials-3526232-supplementary.pdf]

Supporting Information

Oxygen vacancy modification MIL-125(Ti) promotes CO_2 photoreduction to CO with near 100% selectivity

Hangmin Xu[1], Hao Song[1], Jian Xu[1], Xiaozhi Wang[1,2], Xingwang Zhu[1,*]*

[1] College of Environmental Science and Engineering, Institute of Technology for Carbon Neutralization, Yangzhou University, Yangzhou 225009, China

[2] Jiangsu Collaborative Innovation Center for Solid Organic Waste Resource Utilization, Nanjing 210095, China

*Corresponding author: xzwang@yzu.edu.cn (X.W.); zxw@yzu.edu.cn (X.Z.)

Characterization of the photocatalysts

The crystal structure of the catalysts was analyzed using powder X-ray diffraction (XRD) on an AXS D8 ADVANCE Bruker diffractometer with Cu Kα radiation. Electron paramagnetic resonance (EPR) analysis was performed using an A300-10/12 Bruker spectrometer. The microstructure, morphology, elemental mapping of catalysts, were analyzed using field emission scanning electron microscopy (FESEM, S4800II, Hitachi) and high-resolution transmission electron microscopy (HR-TEM, Tecnai G2 F30 S-TWIN, USA). The chemical states of the elements on the catalyst surface were characterized using X-ray photoelectron spectroscopy (XPS, ESCALAB250Xi, Thermo Fisher Scientific). Ultraviolet photo-electron spectroscopy (UPS) was tested with an unfiltered HeI (21.22 eV) gas discharge lamp at a total instrument energy resolution of 100 meV. The nitrogen adsorption-desorption curves over catalysts were determined using a fully automated specific surface area analyzer (ASAP2460, Micromeritics Instrument). The *in situ* FTIR spectra were obtained by a Thermo Scientific Nicolet iS50. The sample was placed uniformly on the substrate lying in the center of the designed reaction cell. High-purity CO_2 (200 $mL\cdot min^{-1}$) was purified for 20 minutes to remove surface adsorbed substances. The high-purity CO_2 was then continuously introduced into the test chamber through a homemade bubbler at a flow rate of 100 $mL\cdot min^{-1}$ until the raw gas was adsorbed and saturated on the catalyst surface, and then the background was collected under dark conditions. At last, the light was turned on, and the signal was collected through the ATR detector during the reaction. UV-vis absorption spectroscopy was performed by UV-vis-NIR spectroscopy

(Cary 5000, Agilent).

Photoelectrochemical Test

The CHI660E electrochemical system is equipped with a standard three-electrode system for measuring photocurrent and impedance. The 10 mg sample was dispersed in a solution of 950 μL ethanol and 50 μL Nafion. Then, 50 μL of the suspension is cast on the indium oxide (ITO) coated glass and dried on an area of 1 cm × 1 cm. The sample with ITO served as the working electrode, Ag/AgCl was the reference electrode, Pt wire was the counter electrode, and 0.2 M Na_2SO_4 was the electrolyte. A 300 W Xe lamp (PLS-SEX300, Beijing Perfectlight) was the light source.

Computational methods

This work implements density-functional theory (DFT) calculations in the Vienna ab-initio simulation package (VASP) and optimizes their structure. The exchange-correlation energy is described using the generalized gradient approximation (GGA) of the PBE exchange-correlation generalization. To achieve an accurate density of electronic states, we integrated the Brillouin-region using a 1 × 1 × 1 Monkhorst-Pack lattice during the iteration process, with a plane-wave cut-off energy of 520 eV.

We performed ion relaxation under the conventional energy (1×10^{-5} eV) and force (0.02 eV/Å) convergence criteria. By testing all possible configurations of $*CO_2$, *COOH, and *CO adsorbed on the MIL-125(Ti) and MIL-125-2H surfaces in the potential active sites, their ground state structures were determined. The configuration with the lowest energy was selected. The free energy was calculated for adsorbed and non-adsorbed gas phase molecules using the equation:

$$\Delta G = E_{total} - E_{slab} - E_{mol} + \Delta E_{ZPE} - T\Delta S$$

where E_{total} is the total energy for the adsorption state, E_{slab} is the energy of pure surface, E_{mol} is the energy of adsorption molecule, ΔE_{ZPE} is the zero-point energy change and ΔS is the entropy change.

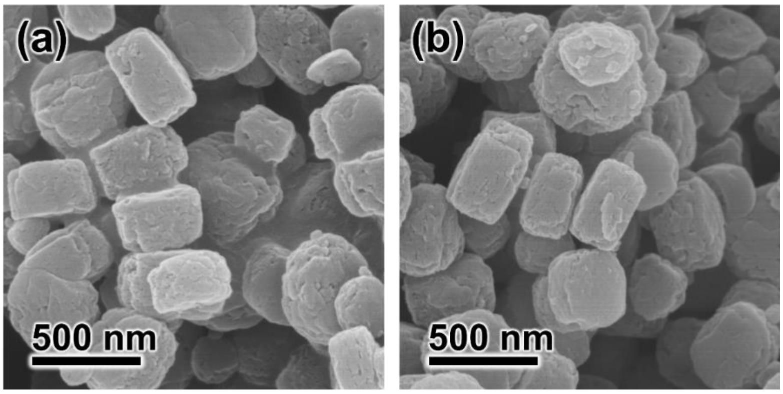

Figure S1. The SEM images of (a) MIL-125-0.5H and (b) MIL-125-5H.

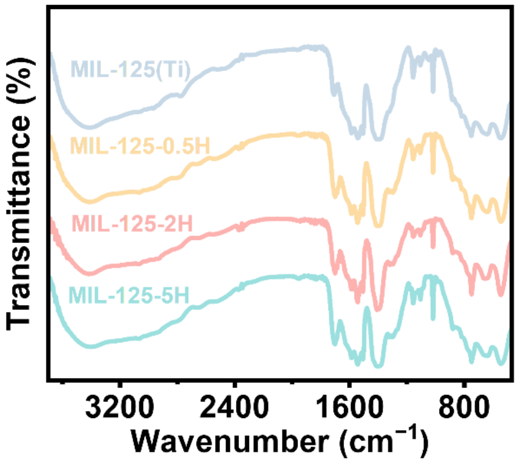

Figure S2. FTIR spectroscopy of the sample.

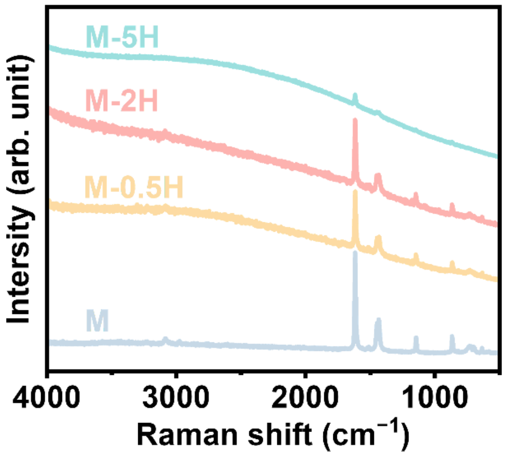

Figure S3. Raman spectroscopy of the sample.

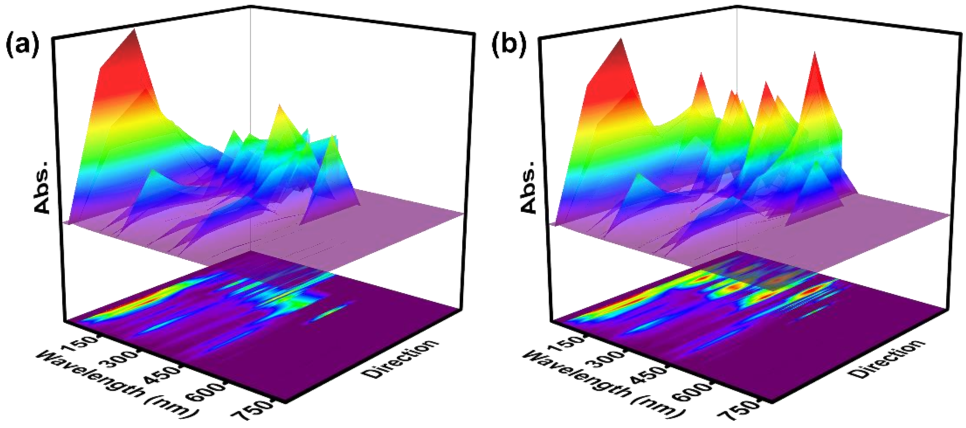

Figure S4. Theoretical calculation of visible light absorption range of (a) MIL-125(Ti) and (b) MIL-125-2H.

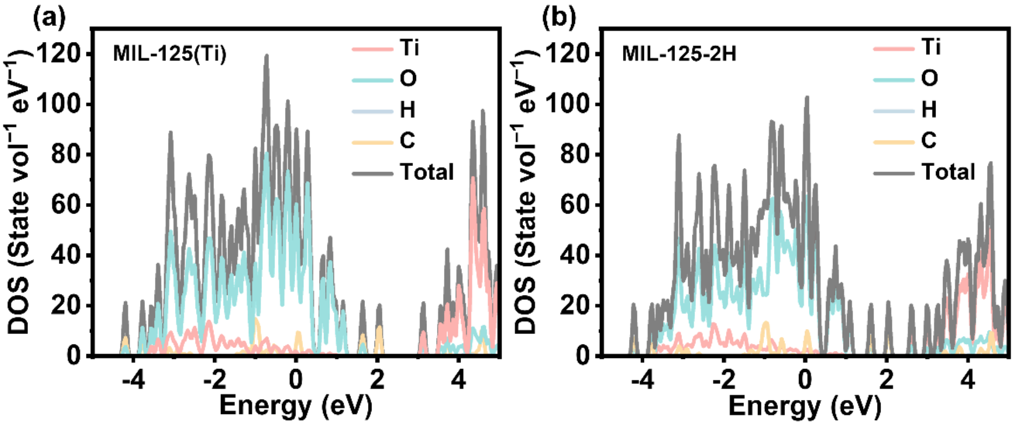

Figure S5. The density of state of (a) MIL-125(Ti) and (b) MIL-125-2H.

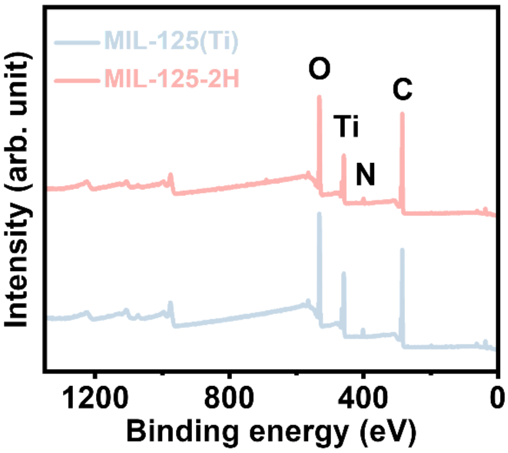

Figure S6. The full XPS spectrum of MIL-125-0.5H and MIL-125-5H.

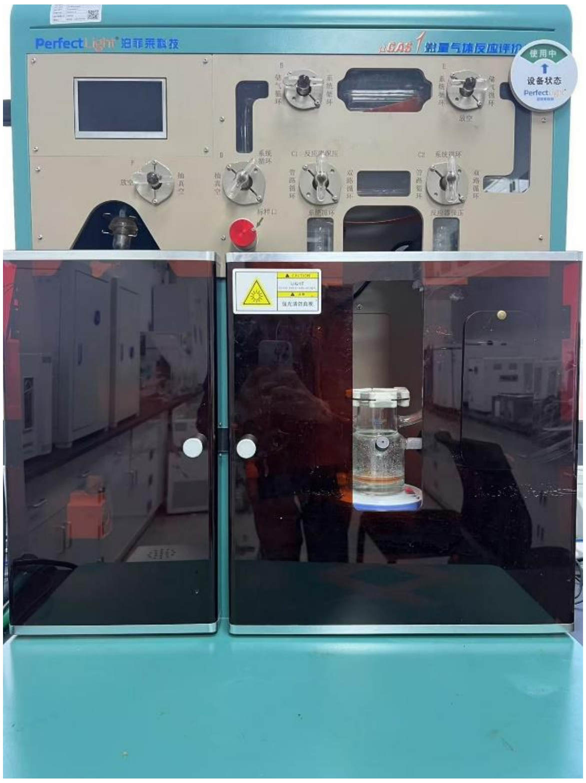

Figure S7. CO_2 photocatalytic reaction system.

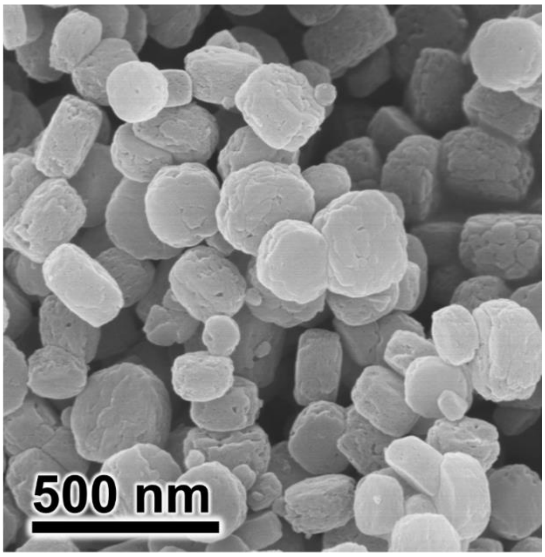

Figure S8. SEM image of MIL-125-2H after stability test.

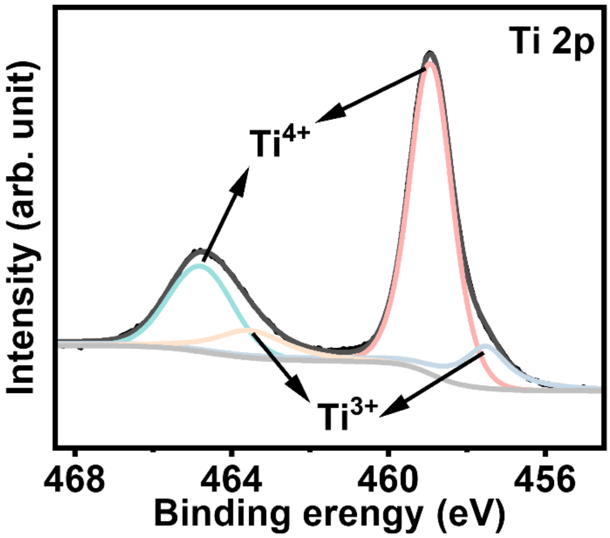

Figure S9. The XPS spectrum of MIL-125-2H after stability test.

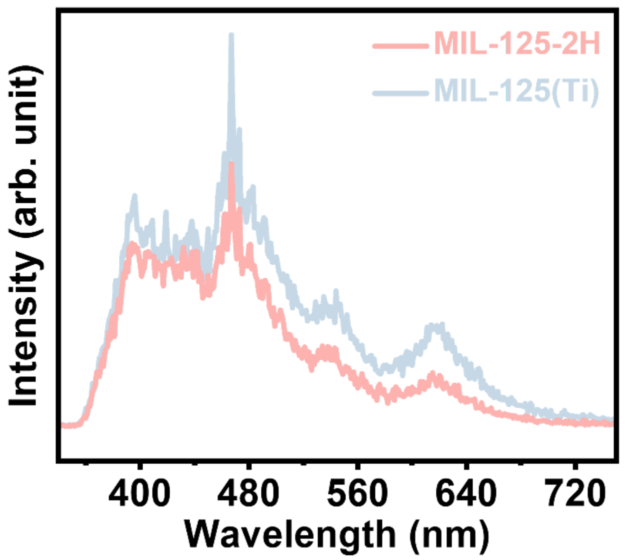

Figure S10. The PL spectrum of MIL-125(Ti) and MIL-125-2H.

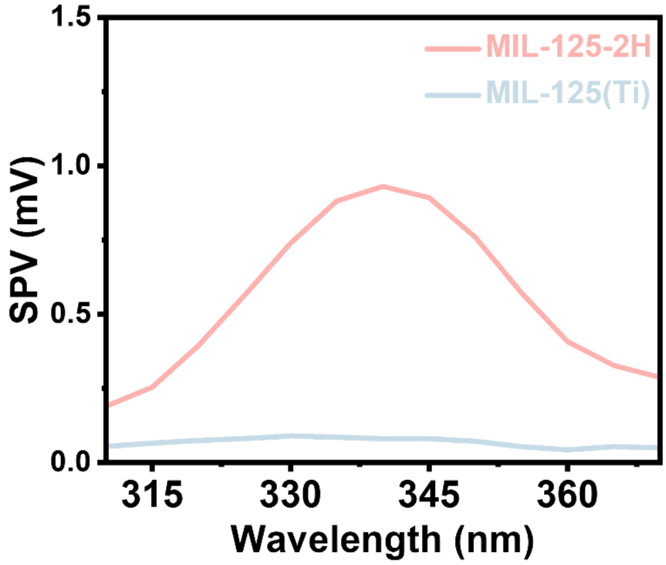

Figure S11. The SPV image of MIL-125(Ti) and MIL-125-2H.

Table S1. The crystallite size of the sample.

Sample	Crystallite size (nm)
MIL-125(Ti)	17.09
MIL-125-0.5H	17.84
MIL-125-2H	25.51
MIL-125-5H	18.19

Table S2. Ratio of Ti^{3+} and T^{4+} content.

Sample	Ti^{3+} (%)	Ti^{4+} (%)	Ti^{3+}/Ti^{4+}
MIL-125(Ti)	18.5%	81.5%	0.23
MIL-125-2H	22.8%	77.2%	0.30

Table S3. The ratio of Ti^{3+}/Ti^{4+} before and after the MIL-125-2H reaction

MIL-125-2H	Ti^{3+} (%)	Ti^{4+} (%)	Ti^{3+}/Ti^{4+}
Before	22.8%	77.2%	0.30
After	23.1%	76.9%	0.30

Table S4. Comparison of the performance of this work with similar literature on photocatalytic reduction of CO_2 to CO.

Sample	Reaction system	CO production rate ($\mu mol\ g^{-1}\ h^{-1}$)	CO Selectivity	Ref.
Bilayer nMOF 2	MeCN, H_2O, TEOA	112.25	100%	[1]
NiCu-MOF	H_2O	12.45	99.0%	[2]
Co-UiO-67	$[Ru(bpy)_3]Cl_2$, MeCN, H_2O, TEOA	3292.50	/	[3]
Cu_2O@Cu@UiO-66-NH_2	TEOA, acetone, H_2O	20.90	38.6%	[4]
NH_2-MIL-125-Ti/WO_{3-X}	H_2O	12.57	/	[5]
Ni_X/TiO_2-Ov	H_2O	22.65	59.0%	[6]
g-C_3N_4/Ov-Ti^{3+}-TiO_2	H_2O	38.40	/	[7]
**This work**	MeCN, H_2O, TEOA	771.22	~100%	/

Reference

[1] J. Liang, H. Yu, J. Shi, B. Li, L. Wu, M. Wang, Dislocated Bilayer MOF Enables High-Selectivity Photocatalytic Reduction of CO_2 to CO, Adv. Mater., 2023, 35: e2209814.

[2] L. Zhang, G. Zhou, G. Chen, H. Wang, Q. Zhao, W. Yin, J. Yi, X. Zhu, X. Wang, X. Ning, Bimetallic NiCu catalyst derived from spent MOF adsorbent for efficient photocatalytic CO_2 reduction, Chem. Eng. J., 2024, 497: 154701.

[3] X. Gao, B. Guo, C. Guo, Q. Meng, J. Liang, J. Liu, Zirconium-Based Metal-Organic Framework for Efficient Photocatalytic Reduction of CO_2 to CO: The Influence of Doped Metal Ions, ACS Appl. Mater. Interfaces, 2020, 12: 24059-24065.

[4] X. Zhao, L. Sun, X. Jin, M. Xu, S. Yin, J. Li, X. Li, D. Shen, Y. Yan, P. Huo, Cu media constructed Z-scheme heterojunction of UiO-66-NH_2/Cu_2O/Cu for enhanced photocatalytic induction of CO_2, Appl. Surf. Sci., 2021, 545: 148967.

[5] H. Jiang, L. Wang, X. Yu, L. Sun, J. Li, J. Yang, Q. Liu, Precise regulation of built-in electric field over NH_2-MIL-125-Ti/WO_{3-x} S-scheme heterojunction for achieving simultaneous formation of CO and H_2O_2 from CO_2 and H_2O, Chem. Eng. J., 2023, 466: 143129.

[6] Z. Li, W. Bai, D. Liu, B. Han, Y. Liang, J. Qi, Preloaded oxygen vacancy conditioning Ni/TiO_2 to enhance photocatalytic CO_2 reduction, Sep. Purif. Technol., 2024, 330: 125250.

[7] Y. Zhang, Y. Wang, Z. Hu, J. Huang, S. Yang, H. Li, High-efficiency photocatalytic CO_2 reduction enabled by interfacial Ov and isolated Ti^{3+} of g-C_3N_4/TiO_2 Z-scheme heterojunction, J. Colloid Interface Sci., 2024, 663: 891-901.
